# The influence of the video assistant referee on the Africa cup of nations football tournaments

**DOI:** 10.3389/fspor.2026.1771811

**Published:** 2026-05-20

**Authors:** Alliance Kubayi

**Affiliations:** Department of Human Movement and Therapeutic Sciences, Tshwane University of Technology, Pretoria, South Africa

**Keywords:** decision-making, fouls, officials, offsides, playing time

## Abstract

This study explored how the implementation of VAR has affected gameplay and referees’ performance during the Africa Cup of Nations (AFCON) football tournaments. The sample comprised all 84 matches played during the AFCON competitions before and after the introduction of the VAR system. The following match variables were recorded: goals, penalties, offsides, fouls, yellow cards, red cards, first-half playing time, second-half playing time, and total playing time. Data were collected from the “Whoscored” website (https://www.whoscored.com). The Mann–Whitney *U* test and a generalised linear model were used to examine match performance variables before and after the introduction of the VAR. The results indicated signiﬁcant (*p* < 0.001) decreases in the number of offsides and fouls after the introduction of VAR. The implementation of VAR resulted in significant (*p* < 0.001) increases in both first-half, second-half playing time, and total playing time. The current findings may have useful ramifications for coaches, players, and sports federations seeking to better comprehend the impacts of VAR in Africa and assist referees in maximising their performance.

## Introduction

Referees are essential in team sports because they are in charge of impartially interpreting and enforcing the rules to ensure that the game is played safely (1, 2). In fact, the referee's role is to uphold the integrity and fairness of the game as it proceeds (3). Because of this, a referee has to make accurate decisions within split seconds during a game. As such, decision-making is frequently regarded as the most crucial skill for officiating effectively (4, 5). Researchers have identified a number of potential factors that could affect the referee's judgement. For example, an incorrect decision can arise because the referee simply did not see the incident (2). Other contextual factors, including in-game pressures from players and coaches, match location, crowd noise, and others, can influence play and bias the referee's decisions (2, 6–8).

During a typical football match, referees need to make roughly 137 observable decisions (9). Referees' decisions are made under time pressure while processing perceptual information (6, 10). Consequently, poor decision-making by referees is a relatively common occurrence that can directly affect the outcome of a game (11) or even a club's revenues (4). To reduce the possibility of referee errors and support their decision making during a game, the video assistant referee (VAR) was first included in the Laws of the Game in 2018/19 (11–13). VAR was implemented to rectify clear and obvious mistakes in potentially game-changing situations, including goals, penalty decisions, direct red cards, and misidentified players, because referees' decisions are not always completely accurate (2, 14, 15). For example, Spitz et al. (2) found that the accuracy of the correct decisions increased from 92.1% to 98.3% following VAR intervention.

To date, research has been carried out regarding the use of VAR in domestic and international football competitions (16). Lago-Peñas et al. (17) investigated how the implementation of the VAR system affected matches in the German Bundesliga and Italian Serie A leagues. The results showed that following the implementation of VAR, the numbers of fouls, offsides, and yellow cards decreased. In contrast, the amount of minutes added to the playing time increased for the first half, but not for the second half. In the Spanish LaLiga, Errekagorri et al. (13) found that two VAR interventions resulted in a somewhat higher total playing duration than one VAR intervention or no intervention. Furthermore, the study indicated that the number of VAR interventions increased in tandem with the number of goals. In a related study, Lago-Peñas et al. (18) reported that the introduction of VAR resulted in a notably lower number of offside calls and a marginally higher number of minutes added to both halves of the game during the Spanish La Liga.

Han et al. (11) found that after VAR was adopted by the Chinese Super League, the home team advantage slightly diminished, the number of offsides and fouls greatly decreased, and playing times for both the first and second halves increased. In the Brazilian Men's Football Championship, the average number of fouls, yellow cards, and obstructions per game decreased following the implementation of VAR (19). Although these studies offer empirical evidence to support VAR's efficacy exclusively in domestic leagues, Kubayi et al. (6) explored how the introduction of VAR has influenced games at FIFA World Cup competitions. The authors discovered that the number of penalties as well as first-half, second-half, and overall playing time all significantly increased following the implementation of VAR. However, after VAR was introduced, there was a noticeable drop in the number of offsides.

From an African point of view, refereeing was marred by major controversies, especially during the 2021 Africa Cup of Nations (AFCON), where many matches had dubious red cards, questionable decisions, and early ending times. This led to widespread criticism of poor officiating standards, with teams and fans expressing their frustration during the tournament (20). Consequently, the Confederation of African Football had to move from only using the VAR in knockout rounds of the 2021 tournament to using it in all matches of the 2023 tournament.

Although VAR in Africa was formally introduced in 2019, no empirical research has examined how its implementation has changed the game on the continent. Given that the use of VAR may raise competition costs (11, 21), it is essential to investigate its effectiveness in the African context. Therefore, the aim of this study was to explore how the implementation of VAR has influenced games and referees' performance during the Africa Cup of Nations (AFCON) football tournaments. It is hypothesised that (i) the amount of time added in both halves and the number of penalties will increase after the introduction of VAR, and (ii) the number of fouls, offsides, yellow cards, and red cards will decrease in games with VAR.

## Methods

### Match sample

The sample comprised all 84 matches played in the 2015 and 2023 AFCON football tournaments, resulting in the examination of 52 matches played with VAR (2023 tournament) and 32 matches played without VAR (2015 tournament).

### Selected performance variables and data collection

The following performance variables that have a direct bearing on the referee's decisions in each game were considered: goals, penalties, offsides, fouls, yellow cards, red cards, first half playing time, second half playing time, and total playing time (6, 11, 17). Data were obtained from the OPTA Company via the “Whoscored” website (https://www.whoscored.com). The company's observational system (OPTA Client System), which is used to gather statistics on football matches, was found to have inter-operator reliability that reached an acceptable level of *κ* (kappa), ICC, *r*, and SEM values (22).

### Data analysis

Data were reported as means and standard deviations. Because results of the Kolmogorov–Smirnov test showed that the match performance metrics were not normally distributed (*p* *<* 0.05), differences between the selected performance metrics for games played with and without VAR were compared using the Mann–Whitney *U* test. Additionally, a generalised linear model (GLM) was fitted for every match performance variable, with the Bayesian information criterion and 95% confidence interval used to assess the goodness of fit. A significance level was set at 0.05 or less. The Cohen's *d* effect size (ES) was grouped and interpreted as small (0.20), medium (0.50) and large (0.80) (23). All statistical analyses were computed using Prism version 10 software (GraphPad, San Diego, California, United States) and the Statistical Package for the Social Sciences version 29.0 (SPSS Inc., Illinois, USA).

## Results

**Figure 1:**
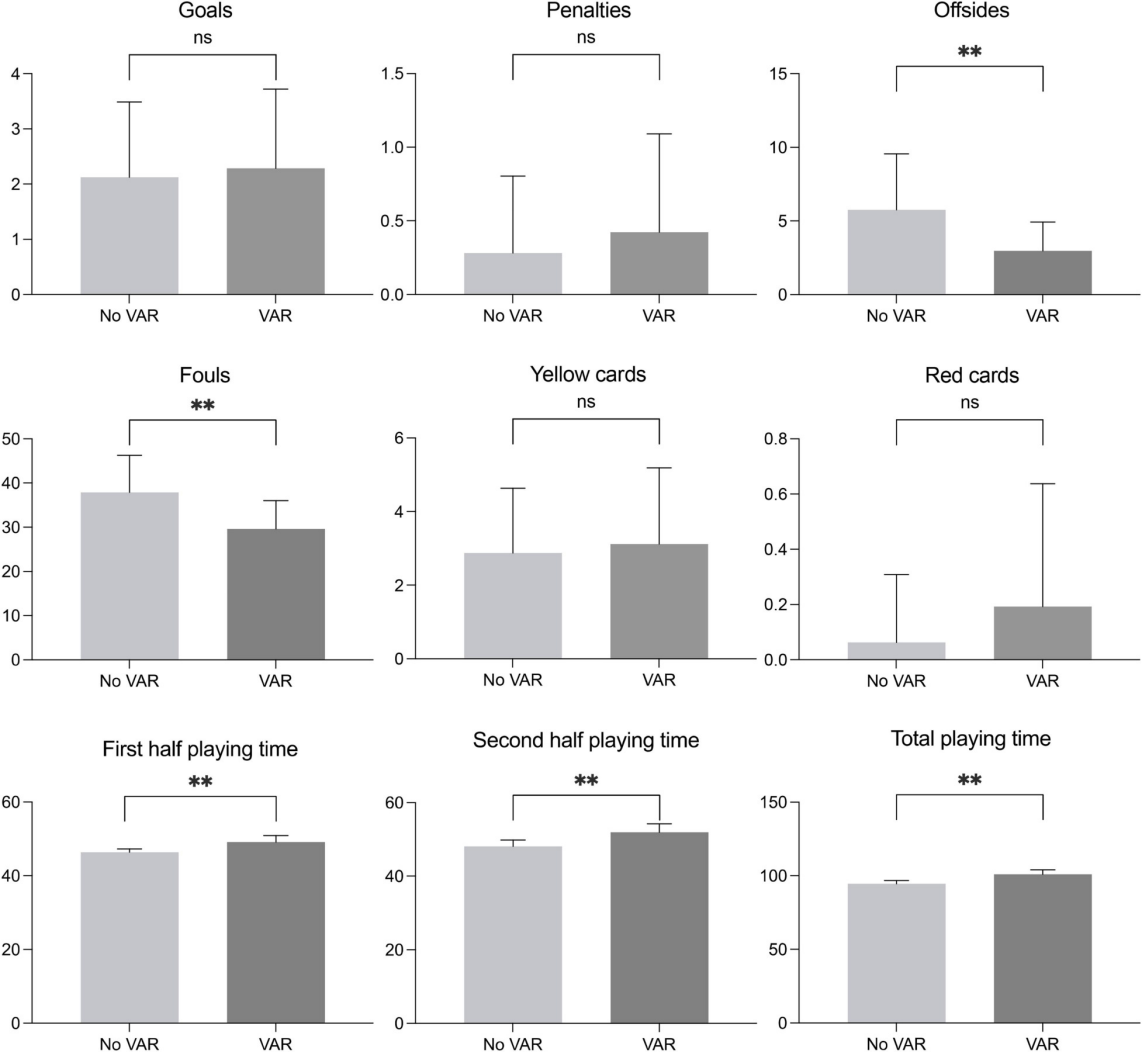
Match performance indicators before and after the introduction of VAR (**p < 0.001; ns, non-significant).

**Figure 2:**
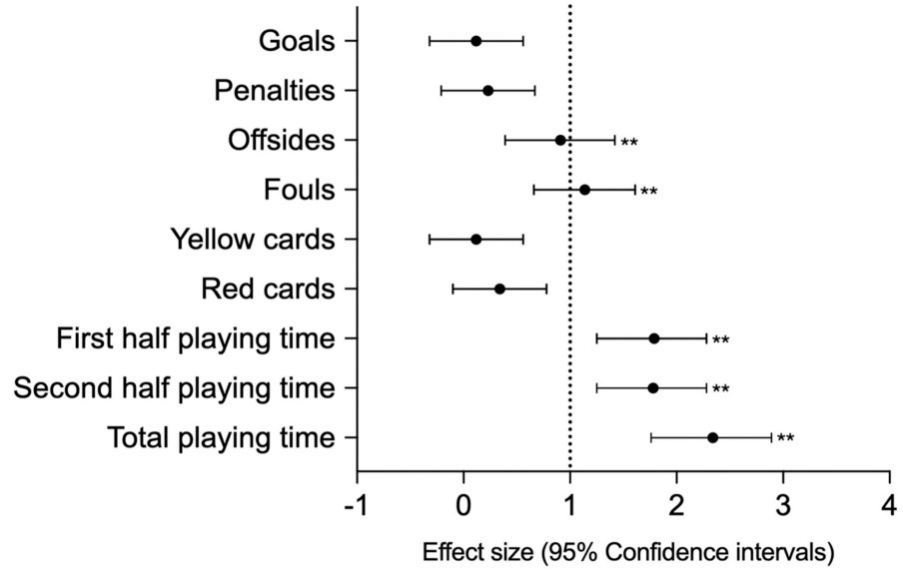
Effect sizes on the match performance indicators before and after the introduction of VAR. Error bars indicate uncertainty in the true mean changes with 95% confidence intervals (**p < 0.001).

Figures 1, 2 show descriptive statistics, ES, and 95% confidence intervals for the selected performance variables at AFCON football tournaments before and after the introduction of the VAR. The results show that there were signiﬁcant (*p* < 0.001) decreases in the number of offsides (*Z* = −3.45, ES = 0.91) and fouls (*Z* = −4.39, ES = 1.14) after the introduction of VAR, as well as significant (*p* < 0.001) increases in first-half playing time (*Z* = −6.58, ES = 1.79), second-half playing time (*Z* = −6.51, ES = 1.78), and total playing time (*Z* = −7.20, ES = 2.34), all with large effect sizes. In addition, the numbers of goals, penalties, yellow cards, and red cards increased, albeit not significantly, after VAR was introduced.

The results of the GLM for each selected performance variable are shown in Table 1. The findings showed that the introduction of VAR significantly affected the number of fouls (*p* < 0.05), offsides (*p* < 0.001), second-half play (*p* < 0.05) and total playing time (*p* < 0.05).

**Table 1:** General linear model for each match performance variable.

Variable	*B*	95% CI		*p*	BIC
		Lower	Upper		
Goals	−0.074	−0.372	0.224	0.626	299.01
Penalties	−0.408	−1.184	0.367	0.302	139.29
Offsides	0.657	0.443	0.871	0.000**	406.41
Fouls	0.247	0.171	0.322	0.000**	584.89
Yellow cards	−0.080	−0.336	0.176	0.538	352.98
Red cards	−1.124	−2.642	0.394	0.147	78.31
First half playing time	−0.058	−0.012	0.006	0.077	492.72
Second half playing time	−0.076	−0.138	−0.013	0.018*	499.94
Total playing time	−0.067	−0.112	−0.022	0.003*	326.52

* *p* < 0.05.

** *p* < 0.001.

## Discussion

This study explored how the implementation of VAR has influenced games and referees' performance during the AFCON football tournaments. The study found that the introduction of VAR resulted in three major findings: (i) the amount of time added in both halves increased, (ii) the number of offsides was reduced, and (iii) the number of fouls decreased. Although the present results support those of previous domestic, continental, and international competitions (6, 11, 13, 17), they also provide new information revealing the positive influence of VAR on certain match performance metrics and referees' decisions in the context of African football.

A key finding of the present study was that the implementation of VAR significantly extended playing time in both halves, resulting in a substantial increase in total match duration, as evidenced by large effect sizes. The current findings contradict those of comparable competition by Bao et al. (24) who reported that the time added by VAR in the UEFA European Championship was limited to 0.8 min in the first half and 0.7 min in the second half, for a total of 1.5 min in the entire game. In the current study, the playing time increased by 2.75 min and 3.77 min for the first and second halves, respectively, for a total of 6.52 min. These results indicated that the game's duration was considerably impacted by the introduction of VAR into the AFCON tournaments. This may be due to the fact that VAR has been used consistently for longer periods of time in European leagues, where officials receive regular training and match exposure. This could have led to quicker decision-making in real time and with fewer interruptions to play. In contrast, VAR is relatively new in many African competitions, and its underutilisation may have led to less “flow” in the decision-making process, resulting into a more significant increase in added time.

A major reason for the increase in playing time is attributed to the suspension of the game when VAR is in use. The ultimate decision in this process must be made after the referee watches the video and consults with the team in the video operation room. This process takes longer than in games played without VAR (6, 11). This could break the game's flow and weaken players' momentum (6) as well as make the game less enjoyable (24). Nonetheless, consistent with previous research (24), it is worth mentioning that the current study did not evaluate other factors directly associated with game flow, such as the number and length of pauses or effective playing time.

Research has indicated an assistant referee's error rate during international football matches of around 13% (25). The authors further noted that wrong offside calls were made twice as frequently in the second half of the game as in the first. Previous studies have demonstrated that such wrong offside calls were probably caused by assistant referees being positioned incorrectly at the precise moment when a decision needed to be made (9, 17, 25). In this light, VAR can play a crucial role in addressing offside calls made by assistant referees. The current study found that the number of offsides calls decreased significantly after the implementation of VAR. This could be attributed to the assistant referees being obliged to delay raising the flag for tight, marginal offside situations if an immediate goal-scoring opportunity exists (14). In the event that an offside occurred before a goal, VAR could examine the situation and reverse the goal (6). Alternatively, teams may have altered their defensive line behaviour and played more cautiously, which would have resulted in fewer offsides because of VAR.

The findings highlight that the number of fouls significantly decreased with the implementation of VAR, substantiating similar findings by Han et al. (11) Because VAR uses at least 12 cameras to monitor the entire stadium, each player's movement trajectory and even their “tiny” points of contact may be seen clearly on the video screen, limiting the players' deliberate fouls (11, 26). This may encourage players to be less aggressive, which could explain the decrease in fouls. Players need to be more cautious of their actions throughout VAR-assisted games, especially when it comes to fouls, tackles, and protests, as the VAR system can assist referees in reviewing the referee's choices through video footage (17).

### Strengths, limitations of the study, and future research

The present findings contribute to a better understanding of how the introduction of the VAR system has changed the game in AFCON football competitions. However, the study has a few limitations that must be considered. The study only included two tournaments before (2015) and after (2023) implementation of the VAR, which limits the generalisations of the findings. However, it should be mentioned that some tournaments (e.g., 2017) were not accessible on WhoScored.com, while in 2019, VAR was only used from the quarterfinals onward. It is also important to note that CAF expanded from 16 to 24 teams in 2019, which may influence the number of teams and changes in team quality, potentially acting as confounding variables when interpreting the results of the current study. Although it was noted that VAR lengthened games, the study did not evaluate effective playing time, a topic that should be investigated in future studies, especially in AFCON competitions. Furthermore, in order to accurately represent the results and reach firm conclusions, future studies should use larger sample sizes for both the pre-VAR (2013, 2015, 2017, etc.) and VAR-assisted tournaments (2021, 2023, etc.).

## Conclusion

This study has explored the impact of the VAR system on match performance indicators at AFCON football tournaments. VAR implementation is associated with a significant shift in match performance indicators, particularly in reduction of fouls and offsides as well as extension of playing time. The findings demonstrate that the increase in playing time is concerning, since VAR frequently disrupts the game's flow and deters players' momentum. Improving VAR protocols may result in faster and more accurate reviews, which might preserve the game's flow while guaranteeing fair and accurate decision-making.

## Data Availability

Publicly available datasets were analyzed in this study. This data can be found here: www.whoscored.com.
